# A Method for Continuous Dual-Offline Payment of Cryptocurrency Based on Asset Credentials

**DOI:** 10.3390/s26103039

**Published:** 2026-05-12

**Authors:** Huayou Si, Yaqian Huang, Guozheng Li, Yuanyuan Qi, Wei Chen, Zhigang Gao

**Affiliations:** School of Information Engineering, China Jiliang University, Hangzhou 310018, China; sshily@cjlu.edu.cn (H.S.); p23030854015@cjlu.edu.cn (Y.H.); p24030854085@cjlu.edu.cn (G.L.); wei.chen@cjlu.edu.cn (W.C.); gaozhigang@cjlu.edu.cn (Z.G.)

**Keywords:** cryptocurrency, continuous dual-offline payment, elliptic curves cryptography, zero-knowledge proofs, smart contracts

## Abstract

With the widespread adoption of cryptocurrencies, the ability to conduct continuous offline payments has increasingly become a critical technological requirement. In network-constrained scenarios, current dual-offline payment technologies are useful for single transactions. However, their limitations in continuous payment scenarios have become increasingly evident, making them unable to meet real-world application needs. This has prompted the industry to demand more urgent innovations in research on continuous offline payment capabilities. To address these challenges, this paper proposes a continuous dual-offline payment system capable of supporting multiple continuous payments. The system integrates elliptic curve cryptography (ECC) and zero-knowledge proof (ZKP) technology to generate secure asset credentials, ensuring both immutability and privacy credentials throughout the offline payment lifecycle. A dynamic credential decomposition mechanism enables the splitting of input credentials into change credentials and receipt credentials, facilitating uninterrupted dual-offline payments between hardware wallets. Additionally, it incorporates a batch verification scheme based on smart contracts, utilizing zero-balance verification and chained hash tracing to ensure payment uniqueness and prevent double-spending attacks, thereby guaranteeing the verifiability and validity of payment settlements. Experimental evaluations demonstrate that the proposed system reduces gas consumption per payment and improves execution efficiency during batch processing, combining high security with strong performance. This research provides a feasible solution for the application of digital currencies in offline scenarios, carrying significant theoretical value and practical significance for driving technological innovation and application expansion in the cryptocurrency field. In addition to cryptocurrency payments, the proposed system is also applicable to IoT and sensor network environments. Many IoT devices operate in disconnected or network-limited areas and require secure micro-transactions. Our dual-offline payment mechanism supports such scenarios, as the main cryptographic operations are lightweight enough for typical IoT hardware. This further extends the practical value of our system beyond traditional cryptocurrency payments.

## 1. Introduction

The rapid development of blockchain technology has also fueled the development of cryptocurrencies [1], which have progressed from conceptual exploration to becoming an integral part of the global financial system [2]. Central banks worldwide have launched numerous central bank digital currency (CBDC) initiatives [3]. Compared to traditional currencies, cryptocurrencies demonstrate significant advantages in reducing payment costs, improving payment efficiency, and optimizing market responsiveness [4]. However, most current cryptocurrency payment systems are heavily dependent on online connectivity, substantially limiting their applicability in special scenarios. In network-constrained environments, online payment models cannot meet user needs. Consequently, offline payment capability has emerged as an essential requirement for numerous practical applications [5]. Thus, dual-offline payment technology has been progressively integrated into digital currency systems [6]. Dual-offline payment technology enables transactional completion when both parties are offline [7], with subsequent blockchain synchronization upon network restoration. This methodology enhances transactional convenience while ensuring payment security. This innovative payment mechanism effectively mitigates the constraints of cryptocurrency implementation, reduces network dependency, and significantly improves the usability and flexibility of cryptocurrencies.

While existing dual-offline payment systems work for single offline transactions, they face significant challenges in supporting multiple consecutive offline payments. First, current cryptocurrency dual-offline payment systems are vulnerable to data manipulation during offline payments, and sensitive information lacks privacy safeguards [8]. Second, existing research has primarily focused on single-payment offline payment verification mechanisms. These studies provide inadequate support for consecutive offline payments. Specifically, they do not properly ensure the integrity, privacy, and verifiability of information during consecutive offline payments [9]. In high-frequency offline payment environments, traditional methods cannot reliably validate sequential transactions. Additionally, current research lacks a systematic assessment of the batch processing capabilities of payment data during dual-offline payment processes [10]. Existing solutions generally lack empirical analyses of the growth trend in gas consumption during batch on-chain payments, hindering accurate scalability performance predictions in actual deployment. Furthermore, existing research has inadequately investigated payment uniqueness verification and on-chain storage mechanisms. As a result, these studies fail to effectively prevent security risks in the final settlement phase of dual-offline payments. Such risks include double-spending and data privacy leaks [11]. Especially in scenarios involving multiple consecutive dual-offline payments, the uniqueness verification mechanisms for payment credentials and data hashes urgently need to be strengthened [12,13,14].

To address these challenges, this paper proposes an integrated architecture for continuous dual-offline cryptocurrency payments. This system encompasses three stages: asset voucher generation and transfer, continuous dual-offline payment, and online batch settlement. These three stages form a complete workflow. Together, they establish a closed-loop system for cryptocurrency offline payments from voucher generation to on-chain verification. For the system design, this paper adopts cryptographic primitives as the foundation, employing elliptic curve cryptography and signature algorithms to generate unforgeable payment credentials. It combines zero-knowledge proofs to achieve payment privacy protection and uses mechanisms such as digital signatures to ensure data integrity and prevent tampering. The system supports multiple credential decompositions and continuous payments in offline mode and employs on-chain signature verification technology to effectively prevent credential reuse and double-spending attacks. The credential decomposition mechanism in this paper differs from Bitcoin’s change output mechanism. Bitcoin’s change mechanism relies on an online blockchain network for transaction confirmation and double-spending detection. Its change outputs are publicly visible and only serve the current transaction. In contrast, the credential decomposition in this paper operates entirely in offline environments, uses zero-sum verification to protect privacy, and the changed credential is designed as the input credential for the next payment, forming a closed loop for consecutive payments. During the online settlement phase, the system provides batch on-chain verification of payment credentials and contract-level global consistency verification, optimizing processing throughput while guaranteeing payment security and high performance. To validate the feasibility and practicality of the system design, this paper implements smart contracts on an Ethereum test network and conducts experimental testing using simulated payment data. Testing content includes trends in gas cost changes during the on-chain process of different batch payments, performance analysis of continuous dual-offline payments, and security assessments of contract verification logic. Experimental results indicate that the proposed system performs well in terms of security and privacy. It also demonstrates outstanding performance in processing efficiency, payment verification costs, and anti-attack capabilities. Therefore, it exhibits strong practical applicability.

The three innovative features of the proposed digital currency continuous offline payment system are as follows:

1. Creatively constructs asset credentials for fund transfers and designs an anti-tampering mechanism for asset credentials to ensure their integrity and authenticity. Elliptic curve algorithms and zero-knowledge proof technology are employed to ensure the privacy and security of asset credentials during their creation and transfer. Elliptic curve algorithms are used to generate key pairs and implement digital signatures for constructing asset credentials. The computational difficulty of the elliptic curve discrete logarithm problem and the integrity verification mechanism of signatures are leveraged to ensure that the information within asset credentials remains unaltered.
2. Creatively designs a credential decomposition mechanism to enable multiple consecutive offline payments between hardware wallets. Input credentials are decomposed into change credentials and receipt credentials, with the original input credentials deleted and the change credentials retained as input credentials for the next dual-offline payment, enabling continuous dual-offline payments.
3. Creatively designs global verification during settlement to ensure that the multiple asset credentials decomposed during settlement are genuine and valid. The payment center verifies the credential signatures to ensure that the decomposed change credentials and receipt credentials comply with the zero-balance equation, combined with the hash of the original credential submitted by the hardware, the asset decomposition chain is traced, and the decomposed credentials are verified through elliptic curve signatures to ensure their authenticity and legitimate origin.

This paper focuses on the construction and verification of cryptocurrency systems in offline payment scenarios, addressing core technical challenges such as payment continuity, security, and verification efficiency. It proposes a cryptocurrency continuous dual-offline payment system with complete functional closed-loop capabilities and batch processing capabilities and validates its feasibility through experiments.

This paper focuses on the complete closed-loop design of a cryptocurrency continuous offline payment system, concentrating on the three-stage process of online fund transfers, continuous offline payments, and online settlement. It comprehensively employs elliptic curve cryptography, zero-knowledge proofs, and blockchain smart contract technology. These techniques systematically address the challenges of payment security, privacy, and efficient verification in environments without network connectivity. Section 1 of this paper outlines the research background and significance, clearly defining the problem statement, research objectives, and innovative points. Section 2 reviews the latest research progress and technical foundations in related fields such as dual-offline payments, anti-double-spending mechanisms, and smart contract security. Section 3 presents the system design from an overall architectural perspective. This design focuses on the collaborative operation of the payment center and hardware wallet. It details the construction of asset vouchers, the continuous offline payment process, batch settlement contracts, and protective mechanisms. Section 4 evaluates the system’s functionality and performance using simulated payment data. It quantitatively analyzes gas consumption and time consumption across different batches and stages. It also visually presents core experimental results through charts. Section 5 discusses the research findings comprehensively based on experimental data. It then assesses their practical value in enhancing the usability, security, and privacy protection of offline payments. Finally, it summarizes the contributions of this paper and identifies its limitations.

## 2. Related Work

Dual-offline payment technology allows users to complete payments without an internet connection, which is particularly critical for payments in areas with limited network coverage, extreme environments, or emergency scenarios. In recent years, multiple dual-offline payment mechanisms have been proposed to enhance the usability of cryptocurrency in offline environments [15,16]. Related research has primarily focused on ensuring that both parties can complete a one-time offline payment while offline and achieve effective synchronization and settlement once the network is restored. Aboulaiz [7] evaluated various global use cases for offline digital payments. Currently, all offline digital payment systems ultimately require internet connectivity for clearing and settlement and can only perform single- or dual-offline payments. However, this approach faces significant challenges related to payment continuity and security, particularly concerning privacy breaches, payment data tampering, and double-spending.

Currently, there are numerous methods for researching and implementing dual-offline payment systems. Various offline payment methods have been discussed and studied in relation to issues such as privacy leaks, tampering with payment data, and double-spending. Thakur [17] proposed a real-time peer-to-peer payments system using a blockchain-based offline channel, leveraging this approach to circumvent scalability issues and enable rapid real-time payments. San [18] proposed an NFC-based privacy-preserving offline mobile payment protocol that uses group signatures to facilitate transactions in offline environments, thereby meeting both security and privacy requirements. Michalopoulos [19] examined privacy and compliance design in CBDC offline payments. He categorized privacy design for offline CBDCs and their corresponding technical building blocks. He also outlined the commonalities and differences between offline and online payment models. Abla [20] proposed a fair and privacy-preserving data payment protocol based on blockchain technology. By combining probabilistic methods with fully homomorphic encryption, the protocol ensures fairness in payments and enables more flexible verification of data validity. Jie [21] proposed a secure and flexible offline payment protocol based on blockchain. The protocol utilizes on-chain smart contracts and offline wallet interactions to establish resilience to intermittent on-chain connections, achieving flexible and trustworthy computing through the use of platform-independent trusted execution environments (TEEs) and open payments. Wu [22] proposed a high-performance smart contract model based on trusted execution environments (TEEs) called Trust-Chain. The aim is to leverage the secure execution environment provided by TEEs to protect the privacy of smart contract code and user data, enabling smart contracts to run securely and privately, thereby enhancing system performance. Fang [23] conducted a comprehensive investigation of cryptocurrency payment systems, concluding that the security of cryptocurrencies is founded on cryptographic principles. Igboanusi [24] proposed an electronic payment architecture named “pure wallet” (PW), which extends the concept of blockchain cryptocurrency to offline payments. This architecture utilizes blockchain and smart contracts to enable financial transactions without an immediate internet connection. Wang [25] proposed a secure and efficient payment solution for multiple offline transactions. The scheme relies on the master public key property of Hierarchical Deterministic (HD) wallets to generate the key pair for the offline wallet (MOBT). Additionally, an interactive signature protocol is used to protect the MOBT from cryptographic attacks during offline payments. Chow [26] analyzed privacy protection issues in cryptocurrency payments. Ivanov [27] proposed a blockchain-based offline payment system called Volga Pay. It designed a novel payment protocol through the combination of polynomial multi-hash chain micropayment channels and blockchain grafting. This protocol requires neither the collection nor the storage of any sensitive data. As a result, it mitigates security threats to system participants. This approach achieves substantial improvements in security, operational efficiency, and cost reduction. Dmitrienko [28] proposed a scheme for secure Bitcoin payments in offline scenarios. The method utilizes offline wallets to verify Bitcoin validity in disconnected environments and incorporates multiple innovative security mechanisms to prevent double-spending. Takahashi [29] et al. proposed a rapid offline payment scheme relying on tamper-resistant wallets manufactured by trusted providers. A segment of the main blockchain serves to validate the legitimacy of pre-loaded coins for the wallet. Li [30] proposed an offline-delegable cryptocurrency system, called Delega Coin, which employs a trusted execution environment (TEE) as a decentralized “virtual agent” to thwart malicious attacks, enabling owners to conduct offline payments without blockchain network interaction. Kurt [31] implemented offline LN payments over wireless networks, called LN Mesh, facilitating local transactions without internet connectivity. The system successfully executes offline LN payments via Bluetooth Low Energy and WiFi while introducing a minimal connected dominating set and unified spanning tree-based channel allocation method to handle high-user-density scenarios. Yang Bo et al. [32] proposed an efficient dual-offline anonymous payment scheme specifically designed for mobile platforms based on trusted execution environments and secure element technology. Yang Bo et al. [33] also proposed DOPS, a feasible dual-offline payment scheme suitable for electronic wallets of two equivalent mobile device users. It constructs the security architecture of mobile devices using only the secure element (SE), while avoiding double-spending, forgery, relay attacks, and other issues. Yang et al. [34] proposed DOT-M to address the practical needs of mobile users for offline payments using central bank cryptocurrency. This scheme meets the practical needs of mobile users for offline payments using central bank cryptocurrency in terms of both security and efficiency. Although existing schemes have made some progress in terms of security and flexibility, they still suffer from issues such as high computational complexity and insufficient privacy protection. Cecchetti et al. [35] proposed combining blockchain with a trusted execution environment (TEE) to enhance privacy protection and payment verification security, but it is primarily applied in online environments and has limited support for offline continuous payments. Existing research has proposed various technical approaches to enhance the security of dual-offline payments. Ahamad [36] and Ansaroudi [37] utilized NFC-based near-field communication technology and hardware wallet mechanisms with built-in secure chips to enable offline payments. Caudevilla [38] and Huibers [39] proposed schemes based on blind signatures and verifiable encryption to enhance payment non-repudiation and repudiation resistance.

In summary, current approaches primarily focus on the privacy of offline payments, the correctness of payment signatures, the immutability of payment records, and double-spending prevention mechanisms for individual payments. These studies have made some progress in achieving the feasibility, security, and privacy protection of dual-offline payments. However, most of this work has concentrated on single offline payment scenarios. As a result, they lack support for the more common scenario of multiple consecutive offline payments in actual systems. In real-world applications, users may complete multiple payments while offline for an extended period, only synchronizing them to the blockchain upon reconnecting to the network. During such consecutive offline payment processes, the mechanisms for generating and verifying payment credentials face heightened requirements for security strength and uniqueness. Without effective mechanisms to prevent duplicate issuance, credential forgery, or sequence tampering, the system would be exposed to severe double-spending attacks and payment tampering risks. Furthermore, when consecutive payments occur between multiple offline nodes, existing solutions struggle to ensure the consistency of digital currency payment sequences, the integrity of the state chain, and the security verification of the entire payment process. Additionally, current methods generally lack support for batch payment processing and efficient on-chain verification mechanisms, resulting in a disconnect between off-chain signing and on-chain verification, which hinders improvements in overall system performance.

To address this issue, this paper proposes and implements a system architecture that supports multiple consecutive dual-offline payments. By combining zero-knowledge proof technology and elliptic curve algorithms, the system enables secure consecutive offline payments, thereby addressing the shortcomings of existing offline payment systems in terms of continuity, security, and scalability. As shown in Table 1, existing systems lack support for consecutive payments and batch settlement, while our system addresses these aspects at the design level. Under the condition of no persistent network connection, the system designed in this paper can ensure that payment data is not tampered with. It can also continuously generate multiple payments. Finally, it guarantees the validity of their verification during the subsequent on-chain process. These design features offer a potential solution for improving the security and practicality of dual-offline payments.

## 3. System Design and Implementation

### 3.1. Offline Payment Architecture Design Based on Asset Certificates

#### 3.1.1. Concept of Credentials

Asset credentials are the core medium enabling continuous dual-offline payments in this system. They are encrypted asset proofs. Each credential contains essential information, including the transaction amount, the holder’s identity identifier, the payment center’s signature, a unique hash value, and a random number to prevent replay attacks. The credential structure is constructed using elliptic curve cryptography (ECC), leveraging the commutative and associative properties of elliptic curve point addition and scalar multiplication. The secp256k1 curve is primarily used as the elliptic curve base type. Initial random points G and H are obtained by randomly generating private keys and calculating their corresponding public keys. This process ensures all subsequent signing and verification operations are executed on standardized elliptic curves, thereby providing cryptographic security guarantees at the protocol level.

Mathematical principles:

1. Commutative and associative properties of elliptic curve addition and scalar multiplication: For any scalars (k,j), the following holds:
  (1) (k+j)×H=k×H+j×H.
  where H is a point on the elliptic curve.
2. Hash operation: SHA256(abc) denotes the hash calculation of abc using the SHA256 algorithm. The symbol “|” in (A|B|C) indicates concatenating A, B, and C in order.

The lifecycle of a credential consists of three stages: the payment center online transfer stage, the continuous dual-offline payment stage, and the final online settlement stage. Each stage incorporates a credential verification mechanism to ensure the security and validity of credentials throughout their circulation, balancing the convenience of offline payments with the security requirements of a digital currency system. At each stage, the Elliptic Curve Digital Signature Algorithm (ECDSA) ensures the integrity of credential content and the authenticity of its origin. A unique identifier is generated using a cryptographic hash function, preventing the credential from being copied or forged. Additionally, the secure chip embedded in the hardware wallet provides physical-level protection, with any unauthorized disassembly attempt triggering automatic data self-destruction. During continuous dual-offline payments, the credential supports dynamic decomposition. When a payment is required, the original credential can be split into two parts: a receipt credential (transferred to the recipient’s wallet) and a change credential (retained in the payer’s wallet). This design allows a single credential to support multiple consecutive offline transactions, while the payment center’s global hash record prevents double-spending.

The core of the proposed method is a signature-based challenge–response protocol designed to verify payment validity without exposing data. This method possesses the zero-knowledge property of verifying truth without revealing secrets, but its implementation differs from general-purpose zero-knowledge proof arithmetic circuits such as zk-SNARKs. Instead, it is realized as a specific cryptographic commitment scheme. The protocol uses elliptic curve cryptography to construct a commitment to the transaction amount. The payer proves ownership of the funds and satisfaction of the zero-sum equation by generating a valid digital signature over the hashed transaction data. This allows the verifier (the payee) to be convinced of the transaction’s validity while being computationally unable to extract the underlying values or the private key, thereby achieving privacy protection in offline scenarios.

#### 3.1.2. System Architecture

**Figure 1:**
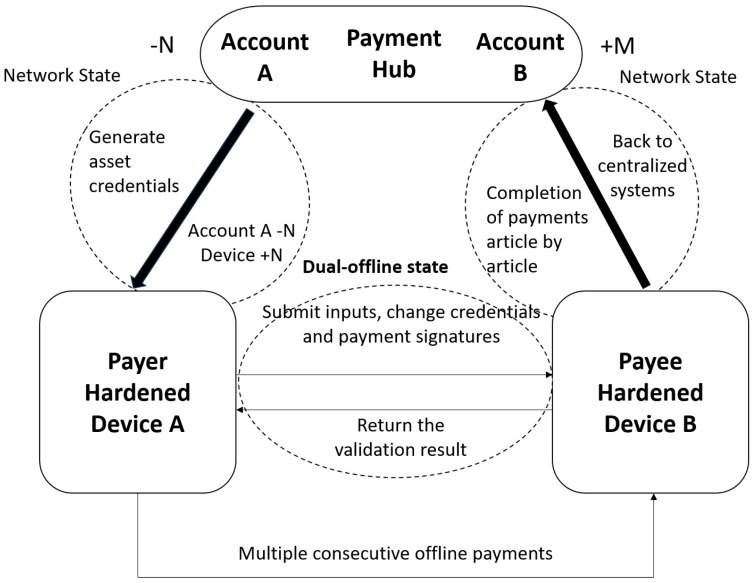
The basic process of cryptocurrency payment.

This paper constructs an offline cryptocurrency payment system comprising two components: a payment center and a hardware wallet, as illustrated in Figure 1. The payment center serves as the accounting core of the payment system, responsible for creating and validating asset credentials, managing payment registration for hardware wallets, and settling payment data back to the central system. The hardware wallet serves as the principal payment instrument in the offline payment system, implementing continuous payment functionality in disconnected states. The complete cryptocurrency payment process is divided into three sequential stages: the online transfer phase, the continuous dual-offline payment phase, and the online settlement phase.

#### 3.1.3. Credential Transfer Process

1. Online transfer phase. The payment center creates the asset credential, then transfers the generated asset credential to the hardware wallet while online. At this point, the corresponding amount is deducted from the payment center’s account and added to the hardware wallet’s funds. The detailed calculation process is shown in Equations (3)–(13).
2. Continuous dual-offline payment phase. The payer (holding wallet A) submits the asset credential (input credential) from hardware wallet A to hardware wallet B for the payee (holding wallet B) in an offline state, completing the dual-offline payment. This process enables multiple consecutive offline payments. The payer’s hardware wallet A decomposes the asset credential (input credential), generating a change credential that is retained in hardware wallet A as an input credential for the next transaction; hardware wallet B generates a receipt credential during the dual-offline payment process; finally, multiple consecutive offline payments generate multiple receipt credentials, which are temporarily stored in hardware wallet B while offline. The detailed calculation process is shown in Equations (14)–(21).
3. Online settlement phase. In an online state, multiple receipt credentials in hardware wallet B are batch-uploaded to the payment center for settlement. This process verifies the validity of multiple newly generated receipt credentials and ultimately determines whether the entire payment was successfully completed.

Across these three phases, the system must address three key issues:

1. After asset credentials are transferred from the payment center to the hardware wallet, ensure that the content has not been tampered with.
2. During multiple consecutive offline payments, ensure successful transactions while preventing double-spending.
3. After asset credentials in the hardware wallet are settled at the payment center, verify that all receipt credentials generated during multiple consecutive offline payments are authentic and valid.

In cryptocurrency payment scenarios, existing offline payment solutions fail to adequately address the above three requirements. This paper proposes a cryptocurrency offline payment system aimed at achieving secure and efficient cryptocurrency offline payments. The system includes a credential tamper-proof mechanism for asset credentials to ensure their integrity and authenticity. First, the tamper-proof mechanism employs elliptic curve cryptography and zero-knowledge proof technology to ensure the privacy and security of asset credentials during their creation and transfer. Elliptic curve algorithms are applied to generate key pairs and implement digital signatures for constructing asset credentials. The computational difficulty of the elliptic curve discrete logarithm problem and the integrity verification mechanism of signatures ensure that the information within the asset credentials cannot be tampered with. Second, a hardware wallet security protection mechanism is designed, integrating anti-tamper detection and self-destruction functions into the hardware wallet, which trigger data erasure upon tampering. Third, a signature verification mechanism is designed, generating a unique hash during asset credential creation and combining dual-signature verification to ensure the integrity of the credential, thereby verifying the authenticity of the information within the asset credential. The system design includes a credential decomposition mechanism to enable multiple consecutive offline payments between hardware wallets. During a dual-offline payment, the payer decomposes the input credential in hardware wallet A into a change credential and a receipt credential, deletes the original input credential, and retains the change credential as the input credential for the next dual-offline payment, thereby enabling continuous dual-offline payments. The system incorporates an anti-double-spending mechanism to address the double-spending issue in offline payments. After each dual-offline payment, the original credential is immediately deleted to prevent its reuse. Additionally, the payment center records the hash of all credentials and rejects duplicate submissions, thereby resolving the double-spending issue. The system includes global verification during settlement to ensure that the decomposed asset credentials are authentic and valid during settlement. The payment center verifies the signature of the credential to ensure that the decomposed change credential and receipt credential comply with the zero-balance equation; combined with the hash of the original credential submitted by the hardware, it traces the asset decomposition chain, and the decomposed credentials are verified through elliptic curve signatures to ensure their authenticity and legitimate origin.

### 3.2. Hardware Wallet Design for Continuous Dual-Offline Payment Architecture

**Figure 2:**
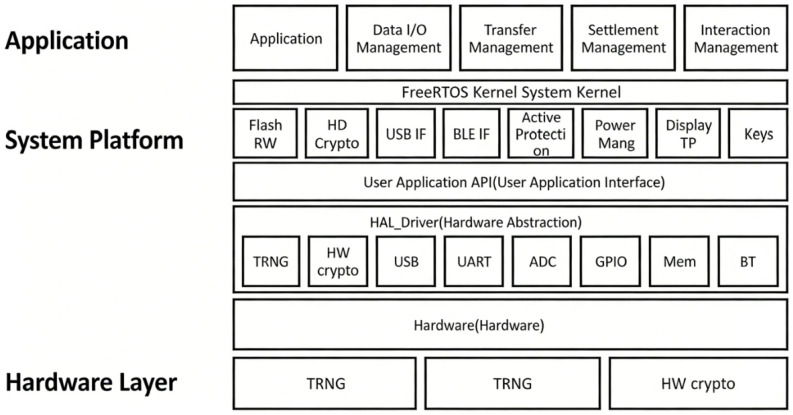
Firmware Architecture Diagram for a Secure Hardware Wallet.

The cryptocurrency payment hardware wallet in the continuous dual-offline payment architecture design is intended to ensure that the continuous dual-offline payment system described in this paper can operate securely and stably on the hardware terminal. This firmware must not only drive the underlying hardware and manage system resources, but also implement core payment-related business logic and proactive security protection. This firmware must handle peripheral device drivers, power management, communication management, and active security protection and, building upon this foundation, implement application-level business logic such as offline transactions and communication with the transaction center. This section presents the architectural diagram of the secure hardware wallet, as shown in Figure 2, which divides the entire firmware into three layers: the hardware interface layer, the system platform layer, and the application layer. The hardware interface layer primarily manages the loading and operation of internal functional modules and peripherals within the chip, including true random number generation, display drivers, external sensor signal detection, and communication interface implementation. The system platform layer, built upon the hardware foundation, provides interfaces for application programs to call, such as display drivers, touchscreen drivers, Flash read interfaces, Bluetooth operation interfaces, power management, and anti-tampering functionality. The application layer implements business logic and functionalities, primarily focusing on features such as offline transactions, fund transfer management, and settlement management.

**Figure 3:**
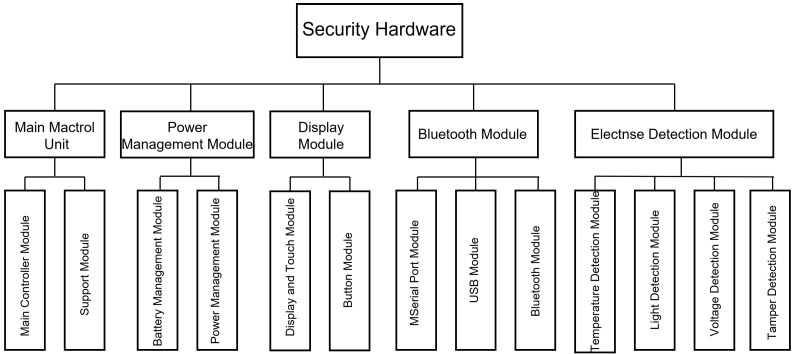
Components of a Hardware Security Wallet.

The core of this device’s design revolves around a dedicated secure element (SE), which provides a tamper-resistant environment and hardware acceleration optimized for SECP256K1 operations, thereby serving as the foundation for secure offline key management and transaction signing. The design of this hardware wallet is divided into a main control unit, a power management unit, an operation interaction unit, an interface unit, and an active protection unit, as shown in Figure 3. The main control unit primarily provides the operating environment for the controller, including essential functions such as the MCU and FLASH memory to maintain system operation. The power management unit, given that the system employs a built-in battery solution, must provide necessary functions, including charging, power supply switching, and voltage conversion. The operation interaction unit handles operations primarily through display and touch input, supplemented by physical buttons; therefore, this module mainly drives the display, touchscreen, and buttons. The interface unit provides three types of interfaces: serial, USB, and Bluetooth modules. Among these, the Bluetooth module serves as the core interface for this solution, facilitating communication required during transactions, while the serial and USB interfaces are used for debugging and other purposes. The active protection unit integrates four functionalities: temperature monitoring, light detection, voltage monitoring, and tamper detection. With the built-in battery configuration, it can immediately detect any hardware damage.

### 3.3. Offline Payment Method Based on Credential Generation

**Figure 4:**
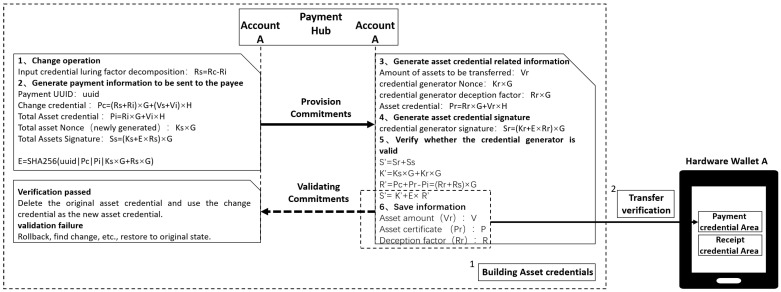
Creation of asset credentials during the network transfer phase.

In a cryptocurrency offline payment system, the online transfer phase undertakes the critical task of initially transferring assets from the payment center to the user’s hardware wallet, providing asset credentials for subsequent continuous dual-offline payments. This phase is completed while online, ensuring the security, integrity, and immutability of credential creation, transfer, and transaction process information. The objective of this phase is to establish initial trusted asset credentials while simultaneously completing the balance transfer and status update between the payment center and hardware wallet, thereby providing a trusted initial credential source for subsequent continuous dual-offline payments and settlement procedures. The entire online transfer process comprises two steps: credential construction and transfer verification, as illustrated in Figure 4.

The payment center constructs asset credentials based on elliptic curve cryptography, utilizing the commutative and associative properties of point addition and scalar multiplication on elliptic curves:

(2) (k+j)×H=k×H+j×H.

where *k* and *j* are private keys, demonstrating that the public key (k+j)×H can be obtained by adding the two private keys, which is equivalent to adding the corresponding public keys of each private key k×H+j×H. In elliptic curve cryptography, selecting a very large number *k* as the private key results in k×H being used as the corresponding public key. Based on the characteristics of elliptic curve cryptography, even if the value of the public key k×H is known, the private key *k* cannot be derived. This paper utilizes the G point and H point on the elliptic curve and the equation structure to construct the form of the asset credential based on the characteristics of the elliptic curve algorithm. Both G and H are random points on the secp256k1 curve, ensuring data complexity and making it difficult to compromise. In the following formulas, let $R_{i}$ denote the blinding factor of the input credential, $R_{c}$ denote the blinding factor of the change credential, $R_{r}$ denote the blinding factor of the receipt credential, and let $R_{s}=R_{c}-R_{i}$ denote the residual blinding factor.

The entire asset credential construction process includes the following six steps:

1. Change operation. First, the payment center receives the transfer request from hardware wallet A and generates the asset credential based on the transfer amount Vs and the system’s internal state. The amount to be transferred to hardware wallet A is calculated from the total assets in the payment center, resulting in a change amount equal to the total assets minus the amount to be transferred.
2. Generate asset information and send it to the credential generation party. The generated content includes the payment UUID, change credential, total asset credential, and total asset nonce. Calculate the credential hash value based on the asset information.
  (3) $$E=\mathrm{SHA}256(\mathrm{uuid}|P_{c}|P_{i}|\left(K_{s}+R_{r}\right)\times G)$$
  Thus, the total asset signature is generated:
  (4) $$S_{s}=\left(K_{s}+E\times R_{s}\right)\times G$$
3. Generate asset credential-related information. This includes the amount of assets to be transferred Vr, the credential generator’s Nonce, and the credential generator’s spoofing factor Rr. Based on this information, the asset credential is generated as follows: credential
  (5) $$P_{r}=R_{r}\times G+V_{r}\times H$$
4. Generate the asset credential signature. Based on the credential hash value calculated in the previous step, generate the signature for the asset credential to be transferred:
  (6) $$S_{r}=\left(K_{r}+E\times R_{r}\right)\times G$$
5. Verify the validity of credential generation. Based on the total asset signature
  (7) $$S_{s}=\left(K_{s}+E\times R_{s}\right)\times G$$
  The asset credential signature is
  (8) $$S_{r}=\left(K_{r}+E\times R_{r}\right)\times G$$
  The signature for the transfer process is $S^{\prime }$:
  (9) $$S^{\prime }=S_{r}+S_{s}=\left(K_{s}+K_{r}\right)\times G+E\times \left(R_{r}+R_{s}\right)\times G$$
  (10) $$K^{\prime }=K_{s}\times G+K_{r}\times G=\left(K_{s}+K_{r}\right)\times G$$
  Because the zero-balance calculation of the amount yields
  (11) $$R^{\prime }=P_{c}+P_{r}-P_{i}=\left(R_{r}+R_{s}\right)\times G$$
  When the asset credential issuer verifies
  (12) $$S^{\prime }=K^{\prime }+E\times R^{\prime }$$
  (13) $$S^{\prime }=S_{r}+S_{s}=\left(K_{s}+K_{r}\right)\times G+E\times \left(R_{r}+R_{s}\right)\times G$$
  If the equation holds, it indicates that the constructed asset credential is valid.
6. Save information. The transferred asset amount, asset credential, and spoofing factor are stored in the payment center.

After the payment center constructs the asset credential, it performs the transfer operation with hardware wallet A while connected to the network. At this point, the payment center sends the asset credential information package to be transferred to hardware wallet A, which includes the transferred asset amount, asset credential, and spoofing factor. Upon receiving the asset credential, hardware wallet A immediately performs integrity verification on the credential information, confirming the hash value; verifies whether the signature equation holds; checks the uniqueness of the credential’s UUID to ensure it has not been reused; and validates the correctness of the asset amount field and structure. If the constructed asset credential passes verification, the hardware wallet treats the current asset credential as a valid credential, completes the status update, deletes the original asset credential, and uses the change credential as the new asset credential; the asset credential is transferred to the payment credential area of wallet A, and the wallet balance is increased by the asset amount. If verification fails, a rollback operation is triggered to restore the system to its original state.

The online transfer phase achieves the first trusted interaction between the payment center and the hardware wallet, completing the generation, signature authentication, transmission, and local verification of the asset credential. In this phase, the system ensures the integrity, non-forgibility, and uniqueness of credential data, providing a trusted foundation for subsequent offline payments and final settlement.

### 3.4. Continuous Dual-Offline Payment Method

**Figure 5:**
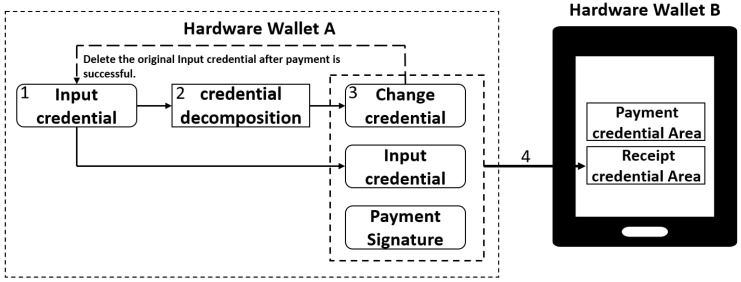
Core principles of multiple consecutive offline payments.

In an offline payment system, the continuous dual-offline payment phase serves as the core process for implementing multiple offline asset transfers between hardware wallets. This phase enables consecutive payment operations in completely offline environments, representing the system’s key operational mechanism when disconnected from the network. To guarantee payment continuity and security, our system designs a comprehensive “continuous dual-offline payment” workflow. The process begins when the payer initiates transactions through hardware wallet A under fully offline conditions, performing asset credential decomposition, change processing, receipt credential generation, and signing operations. Ultimately, multiple new asset credentials are delivered to the recipient’s hardware wallet B for storage, preparing for the subsequent settlement phase, as illustrated in Figure 5.

The basic process of continuous dual-offline payments consists of four steps:

1. Prepare the input credential. The input credential originates from either the original credential initially transferred by the payment center or the change credential retained from previous offline payments. Each offline payment identifies the specific input credential to use and immediately deletes it from the wallet after use to prevent reuse and guard against double-spending attacks. The detailed calculation process is shown in Equation (14).
2. Split the credential. The system processes the amount in the input credential, dividing it into two portions according to the current payment scenario: the payment amount to be transferred to the recipient and the remaining change amount. The system then automatically generates and locally stores a change credential. This step includes automatic verification of a sufficient input amount and ensures the equation “payment amount + change amount = input credential amount” holds true, maintaining the asset conservation constraint. The detailed calculation process is shown in Equation (15).
3. Generate new credentials. The payment process generates two new asset credentials—a change credential and a receipt credential. The change credential retains the remaining amount as the next payment’s input credential, stored locally in hardware wallet A (updating its credential records). The receipt credential is created by hardware wallet B based on the change credential and original input credential information, then stored in hardware wallet B’s receiving credential area. The detailed calculation process is shown in Equations (16)–(19).
4. Transaction verification. Hardware wallet B verifies the transaction and transmits the verification result to hardware wallet A via NFC. Successful verification leads hardware wallet A to adopt the change credential as its new input credential, while hardware wallet B temporarily stores the receipt credential as a pending settlement asset. Failed verification triggers a rollback to the pre-transaction state in hardware wallet A. The detailed calculation process is shown in Equations (20) and (21).

Through these four steps, the system successfully implements multiple continuous dual-offline payments. Each payment operation automatically preserves the change credential for subsequent payments, thereby enabling consecutive dual-offline transactions. This process ensures verifiability, immutability, and double-spending resistance for every offline payment through input credential uniqueness, amount conservation verification, signature mechanisms, and credential transmission.

Based on the design analysis above, the formal consistency of continuous dual-offline payments can be verified as follows. Let *n* denote the number of consecutive offline payments performed by hardware wallet A. The process starts with an initial input

(14) $$P_{i,1}=R_{i,1}\cdot G+V_{i,1}\cdot H$$

issued by the payment center. For each subsequent payment j∈{1,…,n}, the credential is decomposed into a change part $P_{c,j}$ and a receipt part $P_{r,j}$:

(15) $$P_{c,j}=R_{c,j}\cdot G+V_{c,j}\cdot H,\;P_{r,j}=R_{r,j}\cdot G+V_{r,j}\cdot H$$

The continuity is maintained through the chain transition $P_{i,j+1}=P_{c,j}$, which implies that the balance and blinding factors are carried forward: $V_{i,j+1}=V_{c,j}$ and $R_{i,j+1}=R_{c,j}$. Since each step follows the asset conservation rule ($V_{i,j}=V_{c,j}+V_{r,j}$), we define the residual blinding factor as $R_{s,j}:=R_{c,j}-R_{i,j}$. This structure leads to the following local balance observation.

**Property** **1** (**Local Balance Relationship**)**.**
*For each individual payment j, the credentials satisfy the following additive relationship:*

(16) $$P_{c,j}+P_{r,j}-P_{i,j}=\left(R_{s,j}+R_{r,j}\right)\cdot G$$

*This can be verified by substituting the credential definitions and applying the asset conservation equation, which eliminates the H terms and leaves only the blinding factor residue associated with the generator G:*

$$\begin{array}{cc} P_{c,j}+P_{r,j}-P_{i,j} & =\left(R_{c,j}+R_{r,j}-R_{i,j}\right)\cdot G+\left(V_{c,j}+V_{r,j}-V_{i,j}\right)\cdot H\\ & =(R_{c,j}+R_{r,j}-R_{i,j})\cdot G+0\\ & =(R_{s,j}+R_{r,j})\cdot G \end{array}$$

*By extending this logic to the entire sequence of transactions, we can derive a global verification identity. Let $R_{\mathrm{agg}}:=\sum_{j=1}^{n}\left(R_{s,j}+R_{r,j}\right)$ represent the cumulative blinding residue.*

**Proposition** **1** (**Global Telescoping Identity**)**.**
*The entire chain of n payments satisfies*

(17) $$P_{c,n}+\sum_{j=1}^{n}P_{r,j}-P_{i,1}=R_{\mathrm{agg}}\cdot G$$

(18) $$\sum_{j=1}^{n}\left(P_{c,j}+P_{r,j}-P_{i,j}\right)=R_{\mathrm{agg}}\cdot G$$

*This identity is derived by summing the local relationships across all transactions. Due to the transition rule $P_{i,j+1}=P_{c,j}$, the intermediate change credentials and the next step’s input credentials cancel each other out in a telescoping manner:*

$$\begin{array}{cc} \sum_{j=1}^{n}P_{c,j}-\sum_{j=1}^{n}P_{i,j} & =\sum_{j=1}^{n}P_{c,j}-P_{i,1}-\sum_{j=2}^{n}P_{c,j-1}\\ & =\sum_{j=1}^{n}P_{c,j}-P_{i,1}-\sum_{j=1}^{n-1}P_{c,j}\\ & =P_{c,n}-P_{i,1} \end{array}$$

*Consequently, the verification requires only the final state $P_{c,n}$, the set of all receipts $P_{r,j}$, and the very first input $P_{i,1}$.*

*To prevent tampering during the offline session, an aggregate signature $S^{\prime }$ is constructed from the payer’s and payee’s signatures ($S_{s,j}$ and $S_{r,j}$) under the challenge $E_{j}$:*

(19) $$E_{j}=\text{SHA256}({\mathrm{uuid}}_{j}\|P_{c,j}\|P_{i,j}\left\|\left(K_{s,j}+R_{s,j}\right)\cdot G\right)$$

(20) $$S^{\prime }=\sum_{j=1}^{n}\left(S_{s,j}+S_{r,j}\right)=\sum_{j=1}^{n}\left(K_{s,j}+K_{r,j}\right)\cdot G+\sum_{j=1}^{n}E_{j}\cdot \left(R_{s,j}+R_{r,j}\right)\cdot G$$

*By defining the aggregated public key $K^{\prime }:=\sum \left(K_{s,j}+K_{r,j}\right)\cdot G$ and the verification residue $R^{\prime }:=P_{c,n}+\sum P_{r,j}-P_{i,1}$, the final verification rule is established.*

**Proposition** **2** (**Verification Condition**)**.**
*A continuous payment chain is valid and accepted by the payment center if and only if there exists a unified challenge E (enforced by deterministic hashing at settlement, i.e., $E_{j}=E$) such that*

(21) $$S^{\prime }=K^{\prime }+E\cdot R^{\prime }$$

*This formulation mathematically guarantees that the payment center only requires the original input $P_{i,1}$, the final change $P_{c,n}$, and the bundle of receipts ${\left\{P_{r,j}\right\}}_{j=1}^{n}$ to verify the entire offline session. By eliminating the need to transmit or verify intermediate change credentials, the system seamlessly maintains global consistency while significantly optimizing on-chain verification efficiency and reducing storage overhead.*

### 3.5. Batch Credential Verification Settlement Method

**Figure 6:**
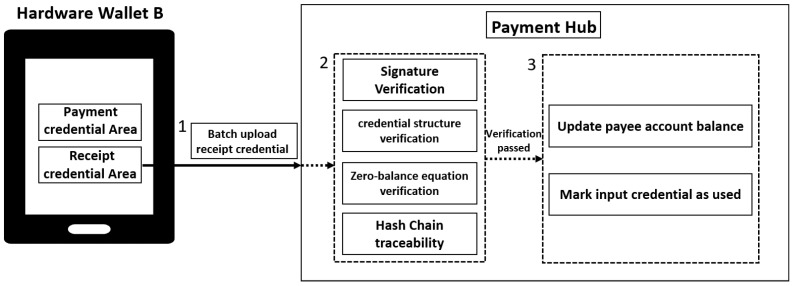
Processing flow in the online settlement phase.

In a cryptocurrency dual-offline payment system, after continuous dual-offline payments are completed, all newly generated receipt credentials are temporarily stored in the receipt credential storage area of the recipient’s hardware wallet B. To finalize the cryptocurrency settlement, the system initiates online settlement processing when network connectivity is restored, uploading all asset credentials generated during previous offline payments to the payment center for centralized verification and processing. The payment center sequentially verifies the credential signatures, structures, amounts, and sources to ensure all payment credentials are authentic and valid, ultimately completing the settlement and status updates. This phase is critical for validating all offline-generated credentials and ensuring their proper mapping to account balance updates, thereby achieving system-level settlement confirmation, as shown in Figure 6.

The entire online settlement process primarily consists of three steps:

1. Batch uploading of receipt credentials to the blockchain. The payee connects hardware wallet B to the payment center while online. At this point, the system automatically submits all pending receipt credentials in chronological order to the payment center in batches. Each uploaded asset credential includes structural fields, signature information, and the hash chain of the original input credential.
2. Verification of the authenticity and validity of receipt credentials. The payment center performs signature verification and credential structure verification on each received receipt credential. Signature verification involves using the payer’s public key to validate the signature in the credential, confirming that the credential’s origin is authentic and not fabricated. During credential structure verification, the system parses the internal fields of the credential to verify whether it conforms to the standard structure definition, preventing missing or forged fields. When the payment center receives multiple receipt credentials requiring settlement, to confirm that the assets in the continuous dual-offline payment process have not been tampered with, the system performs a zero-balance equation verification on the payment chain of the continuous dual-offline payment. During verification, if the equation “payment amount + change amount = input credential amount” is satisfied, and the hash value of the input credential can be found in the payment center’s records as the original hash record, the system deems the payment process complete and valid, and the payment can be successfully settled at the payment center. Additionally, the payment center performs deduplication on the hash values of all settled or pending credentials. If a duplicate hash value is detected in any credential, the system will reject the payment record and mark it as a potential double-spending attempt. The system also uses chained hash relationships to trace the origin and path of each credential, further enhancing its anti-counterfeiting verification capability.
3. Settlement implementation and status update. All verified receipt credentials processed through the payment center are credited to the recipient’s account, completing the asset posting. The status of the corresponding input credential record is marked. Simultaneously, the system generates a complete payment record and settlement credential, concluding the entire dual-offline payment process.

## 4. Security Analysis

To address the security requirements of continuous offline payments and respond to potential adversarial threats, this section establishes a formal adversarial model and provides rigorous security proofs for the proposed system.

### 4.1. Formal Adversarial Model

#### 4.1.1. System Environment and Participant Assumptions

Following the foundational environment definitions established in our previous work [40], the continuous payment system consists of the payment center, hardware wallet A (payer), and hardware wallet B (payee). We assume the payment center is a fully trusted entity, while hardware wallets are semi-trusted (their physical secure elements are trusted, but their human operators or external communication channels may be malicious).

#### 4.1.2. Cryptographic Hardness Assumptions

The security of our formal model relies on the following fundamental assumptions:

- Trusted payment center: The payment center is assumed to be fully trusted. It is responsible for issuing initial credentials, verifying batch settlements, and resolving potential conflicts. The payment center is honest in following the protocol and does not collude with malicious payers or payees. This assumption is necessary for the global verification and batch settlement mechanisms.
- Elliptic curve discrete logarithm problem (ECDLP): Given generator *G* and P=R·G, it is computationally infeasible for any probabilistic polynomial-time (PPT) adversary to compute *R*.
- Random oracle model: The SHA-256 hash function behaves as a true random oracle, guaranteeing collision resistance and pre-image resistance.
- Secure element isolation: The physical hardware enforces strict non-extractability of private keys and atomicity of the internal “decompose-and-delete” credential lifecycle.

#### 4.1.3. Adversary Capabilities

We define an adversary A who operates under the Type I (external eavesdropper) and Type II (internal malicious participant) models. In a continuous payment session of length *n*, A possesses the following formal capabilities:

$C_{int}$  (Intercept): Intercept data transmitted over the offline NFC channel.
$C_{tam}$ (Tamper): Attempt to modify local credential states or storage.
$C_{for}$  (Forge): Attempt to forge Schnorr signatures without the private key.
$C_{rep}$  (Replay): Replay previously captured transaction data.
$C_{cmp}$ (Compromise): Control the device interface to initiate arbitrary offline payments.
$C_{wal}$  (Malicious wallet): Act as a malicious payee/payer attempting to extract additional funds.

#### 4.1.4. Formal Security Goals

The continuous payment protocol must satisfy four formal security goals. For a security parameter λ, the advantage of any PPT adversary A must be negligible:

**Goal** **1** (Balance Privacy): A cannot deduce the actual transaction amount $V_{j}$ or remaining balance from intercepted credentials. ${\mathrm{Adv}}_{A}^{privacy}\left(\lambda \right)\leq \mathrm{negl}\left(\lambda \right)$.
**Goal** **2** (Transaction Integrity): A cannot forge a valid payment credential without detection. ${\mathrm{Adv}}_{A}^{integrity}\left(\lambda \right)\leq \mathrm{negl}\left(\lambda \right)$.
**Goal** **3** (Chain Consistency): A cannot alter any intermediate credential $P_{c,j}$ or $P_{i,j}$ within a continuous chain without failing the global settlement verification. ${\mathrm{Adv}}_{A}^{consistency}\left(\lambda \right)\leq \mathrm{negl}\left(\lambda \right)$.
**Goal** **4** (Anti-Double-Spending across the Chain): A cannot successfully spend the same input credential multiple times across different devices or sessions. ${\mathrm{Adv}}_{A}^{double-spend}\left(\lambda \right)$≤negl(λ).

#### 4.1.5. Threat Scenarios Specific to Continuous Payments

Unlike single offline payments, continuous payments introduce unique threat vectors (TS) that the system must mitigate:

**TS-1** 
(Chain Tampering): A malicious payer alters an intermediate change credential $P_{c,j}$ locally to inflate the input balance for step j+1.
**TS-2** 
(Sequence Attack): An adversary reorders the sequence of continuous payments $\left\{P_{r,1},\ldots ,P_{r,n}\right\}$ to disrupt the settlement logic.
**TS-3** 
(Batch Replay): A malicious payee attempts to batch-upload the same set of valid receipt credentials multiple times during settlement.
**TS-4** 
(Cross-Device Double Spending): A payer clones a valid change credential to another compromised hardware device to initiate simultaneous offline forks.

### 4.2. Security Proofs

#### 4.2.1. Proof of Goal 1 & Goal 2 (Privacy and Integrity)

The basal credential structures $P_{c}=R_{c}\cdot G+V_{c}\cdot H$ are constructed using Pedersen commitments and Schnorr aggregate signatures. As formally proven in previous work [40], Pedersen commitments provide information-theoretic hiding (Perfect Hiding), addressing Goal 1 against $C_{int}$ capabilities. Furthermore, under the ECDLP assumption and the ROM, forging a valid aggregate signature $S^{\prime }$ requires solving the discrete logarithm, which proves Goal 2 against $C_{for}$ capabilities. Since the continuous payment merely iterates these operations, the basal security formally inherits the negl(λ) advantage boundary from the single-payment model.

#### 4.2.2. Proof of Goal 3 (Chain Consistency)

This proof specifically addresses the TS-1 and TS-2 threat scenarios. Assume a Type II adversary A attempts a TS-1 attack by modifying an intermediate credential to $P_{c,j}^{*}\neq P_{c,j}$. According to Theorem 1 (Telescoping Global Verification) derived in Section 3.4, the global verification residue $R^{\prime }=P_{c,n}+\sum_{j=1}^{n}P_{r,j}-P_{i,1}$ strictly depends on the exact telescoping cancellation of intermediate states ($P_{i,j+1}=P_{c,j}$). The introduction of $P_{c,j}^{*}$ breaks this transition constraint, meaning the intermediate terms will not cancel out.

Consequently, during the online settlement phase, the payment center verifies Theorem 2: $S^{\prime }=K^{\prime }+E\cdot R^{\prime }$. Because $R^{\prime }$ is now corrupted and does not match the scalar sum protected by the aggregated signature $S^{\prime }$, the equation will deterministically fail. To bypass this, A must forge the aggregate signature $S^{\prime }$ for the corrupted $R^{\prime }$, which violates the ECDLP assumption. The unified challenge *E* (bound to the chronological UUID hash) further prevents TS-2 sequence attacks. Therefore, ${\mathrm{Adv}}_{A}^{consistency}\left(\lambda \right)\leq \mathrm{negl}\left(\lambda \right)$.

#### 4.2.3. Proof of Goal 4 (Anti-Double-Spending Across the Chain)

This proof addresses the TS-3 and TS-4 threat scenarios. We define the security game where A attempts to duplicate purchasing power.

**Against TS-4 (local offline double-spending)**: The protocol leverages the SE isolation assumption (Section 4.1.2). The SE enforces an atomic hardware-level “decompose-and-delete” routine: once an input credential $P_{i,j}$ is split, it is permanently erased from the non-volatile memory before the next interaction can proceed, nullifying $C_{wal}$ and $C_{tam}$ capabilities.

**Against TS-3 (online settlement replay)**: The smart contract at the payment center acts as the ultimate arbiter. As detailed in Section 3.5, the contract records the deterministic, chronologically linked UUIDs of all settled credentials. If A submits a cloned branch of credentials, the contract’s hash deduplication algorithm will flag the collision in O(1) time and revert the transaction.

Hence, any double-spending attempt is mathematically and structurally thwarted, ensuring ${\mathrm{Adv}}_{A}^{double-spend}\left(\lambda \right)\leq \mathrm{negl}\left(\lambda \right)$.

## 5. Experimental Design and Performance Evaluation

### 5.1. Experimental Environment and Deployment

To validate the feasibility and effectiveness of the proposed cryptocurrency dual-offline payment system, this paper completes smart contract development, system functionality testing, and performance evaluation in a locally deployed environment.

The experiments were conducted on a hardware platform equipped with an Intel^®^ Core™ i5-1035G1 quad-core processor (1.19 GHz) and 8 GB of memory, running the Microsoft Windows 11 operating system (version 22631.4317) on a Dell Inspiron 5493 device (Dell Inc., Round Rock, TX, USA). The system implementation is based on JavaScript, with elliptic curve operations (secp256k1) performed using the Elliptic library (v6.5.4). All random number generation relies on the cryptographically secure Web API crypto.getRandomValues().

1. Development platform and tools. The cryptocurrency dual-offline payment system in this paper uses the Solidity language to write the core contract logic, employs Hardhat as the local contract development and deployment framework, builds a test blockchain environment, and conducts multiple rounds of functionality and performance testing. The selected contract compiler version is Solidity v0.8.28, which offers robust security and computational stability. Additionally, to support offline payment verification and asset credential structure design, the system integrates the ECDSA cryptographic library and SHA256 hash function provided by Open Zeppelin to implement a dual-signature mechanism, verify data integrity, and prevent tampering. The tools and components used in the experiment are shown in Table 2 below.
2. Local blockchain deployment environment. To achieve precise control and batch payment testing, this paper utilizes the local blockchain simulation node provided by Hardhat for deployment. This node can simulate a high-performance operating environment, facilitating repeated execution of batch payment scripts and monitoring of gas fee consumption. The local blockchain is configured as follows: the single-block gas limit is set to 100,000,000 to ensure high-concurrency payment writes; the allowUnlimitedContractSize configuration is enabled to deploy large complex contracts; the payment packaging block time remains the default to simulate confirmation delays in real networks.
3. Smart contract deployment and functional coverage. The proposed cryptocurrency dual-offline payment system mainly deploys two core smart contracts: the payment storage contract and the wallet contract. The payment storage contract supports batch storage of payment data, dual-signature verification of credentials, and asset splitting records; the wallet contract simulates fund transfers and settlements in the dual-offline payment system while maintaining payment credential structures. Contract deployment uses automated deployment scripts written in JavaScript, which are executed via commands on the local network and output contract addresses upon completion for subsequent test calls.
4. Test scripts and data generation methods. To simulate dual-offline payment scenarios, the system designs a batch payment test script supporting multiple functions, including the automatic generation of N offline payment data per test round. Each payment contains the following fields: payment UUID, asset credential Pi, change credential Pc, and signature Ss. The script also simulates credential decomposition and hash chain recording in continuous payment scenarios, performs batch calls to the storeTransactionsBatch() function for on-chain processing, and records gas consumption and execution time for each batch. Test data is generated automatically using local pseudo-random functions and the UUIDv4 library, ensuring data independence and unpredictability.
5. Performance testing. For systematic evaluation of batch on-chain performance, the system implements a console. The following are measured: total gas consumption per payment round, average gas consumption per payment, total execution time, and average execution time per payment.

### 5.2. Functional Validation Testing

To evaluate the feasibility and performance of the designed dual-offline payment system in multiple consecutive payment scenarios, this paper conducts comprehensive experimental verification of the system’s core functionalities. The experiments cover payment batch processing capability, gas cost variation trends, and per-transaction resource usage efficiency, aiming to quantify the system’s operational energy consumption under different payment scales and analyze its performance stability and resource conservation capability during large-scale continuous payment processes.

**Figure 7:**
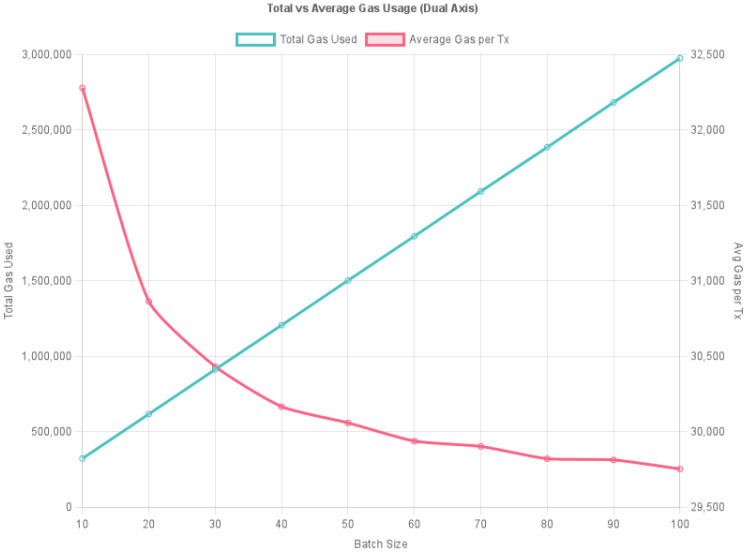
Trends in gas consumption and unit cost under different batch payment methods.

**Figure 8:**
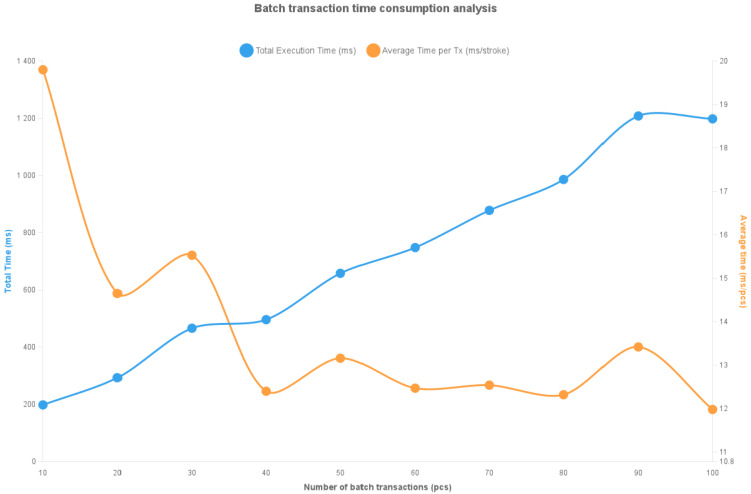
Trends in time consumption and unit cost under different batch payment methods.

The experiment uses batch payment simulation programs written in JavaScript to simulate multiple rounds of offline payment batches of varying scales. By calling the system contract interface storeTransactionsBatch(), the program performs batch storage operations of payment data and records gas consumption and time consumption performance metrics generated by on-chain contract execution. The experimental protocol includes batch on-chain testing of offline payment data with different batch sizes (10, 20, 30, 40, 50, 60, 70, 80, 90, and 100 payments), recording and statistically analyzing gas consumption and execution time for each group. After multiple repetitions of each group, average values were calculated, ultimately generating the dual-axis line charts shown in Figure 7 and Figure 8 to examine the impact of payment quantity on blockchain execution efficiency.

As shown in Figure 7, the line chart consists of two lines, representing the total gas consumption of the system under different payment batches and the average gas consumption per payment, i.e., the average gas consumption per transaction within that batch. Based on the experimental results in Figure 7, the following conclusions can be drawn:

1. The total gas consumption of the system for continuous dual-offline digital currency payments exhibits non-linear growth. As the batch processing scale increases from 10 to 100 transactions, the system’s total gas consumption shows a non-linear upward trend. This indicates that the system has good scalability in its overall processing logic and can reliably handle larger batches of continuous offline payment requests.
2. The proposed continuous dual-offline digital currency payment system operates stably across multiple batch scenarios. The gas consumption results show that as the batch size increases, the gas consumption per transaction decreases from 32,000 to approximately 29,000, representing an overall reduction of about 10%. The decreasing rate slows down and stabilizes as the batch size grows. All batch processing experiments were completed without exception, with stable gas consumption fluctuations and no abrupt spikes or drops. This demonstrates that both the smart contract logic and signature verification process exhibit excellent stability and can adapt to continuous payment workloads of varying scales.

As shown in Figure 8, the line chart consists of two lines, representing the system’s total execution time under different payment batches and the average execution time per transaction, i.e., the average execution time per transaction in the batch. Based on the experimental results in Figure 8, the following conclusions can be drawn:

1. The total execution time shows non-linear growth with batch size. When the batch size increases from 10 to 100 transactions, the total execution time grows from approximately 0.2 s to slightly above 1.2 s, demonstrating a stable upward trend without drastic fluctuations. This indicates the system maintains good thread scheduling and processing overhead control in batch payment scenarios, enabling linear scaling for larger-scale offline payment requests.
2. The average per-transaction execution time decreases significantly and stabilizes. As the batch size expands, the average execution time per transaction decreases from an initial 19 ms to a minimum of 12 ms, representing a 35% reduction. In the 40–100 transaction range, the average execution time remains stable within a narrow fluctuation band of 12–14 ms, demonstrating that batch processing effectively reduces per-transaction execution time.

The combined results of gas consumption and execution time show that the proposed continuous dual-offline cryptocurrency payment system maintains low per-unit gas consumption while significantly reducing average execution time in batch payment mode, verifying its advantages in processing efficiency and scalability.

In conclusion, the experimental validation demonstrates that the proposed dual-offline cryptocurrency payment system maintains excellent operational efficiency and resource cost performance in batch payment scenarios, making it suitable for deployment in high-frequency, multi-round offline payment environments. Moreover, the system’s signature verification and data storage mechanisms consistently exhibited high stability throughout multiple test rounds, providing reliable assurance for subsequent online settlement and security verification.

**Figure 9:**
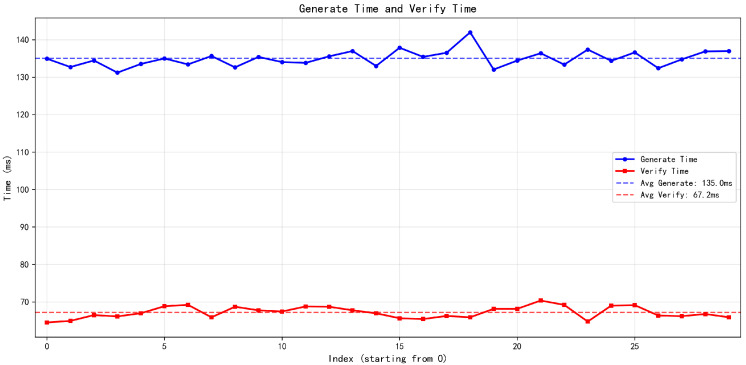
The time required for simulating continuous dual-offline payment tests on hardware devices.

Furthermore, to evaluate the off-chain performance feasibility on hardware wallets, we profiled the local execution latency for the continuous payment steps. To ensure statistical significance, we conducted N = 30 independent profiling trials for the off-chain process, which primarily consists of credential generation and mutual verification. The experimental results demonstrate highly stable performance: the credential generation phase takes an average of 135 ms, while the local verification phase requires approximately 65 ms, as shown in Figure 9. Consequently, a complete continuous offline payment step demands roughly 200 ms of local computation. This latency is well within the standard 300 ms physical tolerance window for typical NFC contactless payments, explicitly confirming that our protocol is highly practical for resource-constrained hardware devices without degrading user experience.

### 5.3. Benchmark Discussion

As summarized in Table 1 in the Related Work section, existing dual-offline payment schemes have significant limitations. Most schemes only support single offline payments and lack the ability to handle consecutive offline transactions. In contrast, our proposed system supports the following continuous offline payment features:

- **Consecutive payment capability:** The system enables multiple rounds of offline payments without requiring re-initialization between transactions. The credential decomposition mechanism automatically generates change credentials that serve as input for the next payment.
- **Batch settlement on blockchain:** After network recovery, all offline receipts can be uploaded and verified in a single batch transaction, significantly reducing on-chain gas costs.
- **Privacy preservation:** Zero-sum verification is integrated to protect transaction amounts and balances during the entire consecutive payment process.
- **Tamper-proof and double-spending prevention:** The asset credentials are protected by elliptic curve signatures, and the atomic “decompose-and-delete” mechanism ensures each credential can only be used once.

It is worth noting that none of the existing works [19,21,22,26] provide reproducible experimental benchmarks or publicly available source code. Due to differences in system architecture, assumptions, and evaluation metrics, a direct experimental comparison cannot be conducted under our current setup. Therefore, we adopt the theoretical comparison shown in Table 1 to demonstrate the design advantages of our system. A comprehensive experimental benchmark comparison under a unified framework is planned as future work.

### 5.4. Engineering Applications of Cryptocurrency Dual-Offline Payment Systems

With the rapid advancement of central bank digital currencies, payment systems are gradually entering a new era of high security and high availability. Therefore, building a dual-offline payment system with offline payment capabilities and robust security protection mechanisms is not only an important supplement to the existing payment system but also a key direction for the future development of intelligent payment networks. The cryptocurrency continuous dual-offline payment system proposed in this paper specifically addresses these requirements by combining zero-knowledge proof technology, elliptic curve algorithms, offline signature verification, and off-chain credential reconstruction mechanisms to establish a digital currency payment system with practical engineering application potential.

In terms of application scenarios, the cryptocurrency dual-offline payment system demonstrates wide applicability in multiple critical situations. For instance, in signal-blind zones such as remote mountainous areas, subway tunnels, and underground shopping malls, traditional QR code scanning or online payment methods often fail. In contrast, this system relies on peer-to-peer communication between hardware wallets and local credential storage/verification mechanisms, enabling multiple consecutive payments under completely offline conditions while maintaining uninterrupted payment experiences. In emergency scenarios where traditional communication infrastructure may be temporarily disabled, the system can maintain basic payment functionality even during power outages and network disruptions, significantly improving the resilience of payment systems.

Technically, the proposed system employs a highly engineered implementation approach. At the contract level, it designs core smart contracts that support credential hash verification, dual-signature anti-tampering, double-spending prevention, and batch settlement. The offline logic layer implements credential decomposition, offline signing, and zero-balance verification through JavaScript scripts. The hardware application layer incorporates tamper-resistant designs with self-destruct mechanisms to ensure credential confidentiality and non-transferability. These technical foundations make the system not just theoretically sound but also practically deployable.

Furthermore, the system achieves both scalability and compatibility with existing infrastructure. Multiple consecutive offline payments can be batch-uploaded when connectivity resumes, with asynchronous settlement and parallel on-chain verification reducing payment center workload while improving throughput. This serves as a paradigm for the future large-scale adoption of cryptocurrency in complex network environments.

The system proposed in this paper can also be applied to IoT and sensor network environments. Many IoT devices often operate in areas without network access or with limited connectivity. Examples include environmental sensors, smart meters, agricultural monitoring devices, wearable devices, and edge nodes in smart cities. These devices often need to make small payments without a stable internet connection. The dual-offline payment mechanism proposed in this paper can well meet this need. This mechanism allows these devices to complete secure micro-transactions without requiring continuous network access. Even after the network is restored, the system can upload multiple offline transactions to the blockchain in batches through a batch settlement mechanism. This ensures payment security while also reducing the cost of on-chain verification. Regarding resource constraints, the main cryptographic operations used in our system are elliptic curve signatures and hash functions. These operations have been proven to be sufficiently lightweight. They can run efficiently on typical IoT hardware. It should be noted that this paper mainly focuses on protocol-level design and functional verification. A detailed performance evaluation on real IoT hardware is planned as future work. This evaluation will include metrics such as computation time, energy consumption, and storage overhead.

Meanwhile, this section discusses the feasibility of the proposed system in practical deployment from three dimensions: cost, user experience, and failure scenarios. In terms of cost, our system uses hardware wallets to store asset credentials and perform signature operations. For high-frequency consecutive offline payment scenarios, the cost of current mainstream hardware wallets is within a reasonable range. In addition, the computational overhead of the zero-sum verification used in this study is reasonably feasible on wallet devices. In deployment, different proof schemes can be selected based on actual security requirements, and part of the computation can be offloaded to the online payment center when network conditions allow. In terms of user experience, the system is easy to operate. Users only need to confirm with one signature to complete a payment. The system supports multiple consecutive offline payments without re-initialization, allowing users to pay continuously just like using cash. Moreover, offline payments generate local transaction credentials and display results in real time. After network recovery, final confirmations are automatically pushed to users, reducing user uncertainty. Therefore, without sacrificing security, our system achieves a good user experience and engineering feasibility. In terms of failure scenarios, when a device is lost, asset credentials and private keys are stored in the secure chip of the hardware wallet, making them inaccessible to attackers. In data desynchronization scenarios, the system assigns a unique credential identifier to each payment to prevent double-spending. The hardware wallet also has built-in credential validity detection. If a user accidentally deletes a change credential, the system will prompt and reject subsequent payments, avoiding invalid transactions.

## 6. Conclusions

This paper addresses current challenges in cryptocurrency offline payments, including difficulties in ensuring continuous payments, underdeveloped double-spending prevention mechanisms, and complex verification of payment credentials. We designed and implemented a cryptocurrency dual-offline payment system emphasizing security and continuity, validating its effectiveness and engineering feasibility through smart contract deployment and multiple experimental verifications. First, we established a dual-offline payment architecture consisting of a payment center and hardware wallets, clearly defining three core phases: online transfer, continuous dual-offline payment, and online settlement. This ensures complete payment lifecycle management capability in offline environments. Second, at the protocol level, we proposed an asset credential construction method based on elliptic curve cryptography and zero-knowledge verification, which innovatively integrates dual-signature structures, credential decomposition mechanisms, and zero-balance verification to achieve credential immutability, payment path traceability, and verifiability of continuous payments. The designed anti-double-spending mechanisms and credential chain tracing significantly enhance system security and attack resistance. Third, we completed full system prototyping using JavaScript and Hardhat, developing smart contracts supporting offline signing, batch on-chain processing, and credential verification. Extensive batch payment testing demonstrated the system’s excellent stability and performance scalability when handling large-scale dual-offline payment data. Experimental results show typical non-linear growth trends in both total gas consumption and average gas per transaction, clearly revealing the system’s resource efficiency and computational costs. Finally, functional verification confirms the system effectively supports multiple consecutive dual-offline payments while ensuring efficiency, high security, and resistance to double-spending, thus providing a practical solution for real-world offline digital currency payments.

In conclusion, our cryptocurrency dual-offline payment system demonstrates significant theoretical value and application potential in architecture design, protocol development, security mechanisms, and engineering implementation. Future research will be deepened across three key dimensions. In terms of security verification, a financial-grade attack testing framework will be established to conduct formal proofs against complex threats such as double-spending hybrid attacks while advancing the prototype development of secure hardware modules. Regarding engineering deployment, practical hardware wallet prototypes and smart contract verification systems will be developed to enable a transition from simulated environments to real-world network validation. For system optimization, efforts will focus on improving the efficiency of communication protocols, enhancing adaptability to high-concurrency payment scenarios, and strengthening hardware resilience against physical attacks. The advancement of these initiatives will progressively solidify the engineering foundation of the dual-offline payment system, providing robust support for its practical deployment in cryptocurrencies and even central bank digital currency applications. The proposed system can also be applied to IoT and sensor network environments. Many IoT devices operate in disconnected or network-limited areas. Our dual-offline payment mechanism enables these devices to perform secure micro-transactions without constant network connectivity. The main cryptographic operations are lightweight enough to run on typical IoT hardware. As discussed in the Introduction, a detailed performance evaluation on real IoT hardware is planned as future work.

## Figures and Tables

**Table 1:** Qualitative feature-based comparison with existing dual-offline payment schemes.

Feature	Paper [19]	Paper [21]	Paper [22]	Paper [26]	Ours
Supports consecutive offline payments	✗	✗	✗	✓	✓
Anti-tampering mechanism	✓	✓	✗	✗	✓
Privacy protection	✓	✓	✓	✓	✓
Batch settlement on blockchain	✗	✓	✓	✗	✓
Experimental benchmark provided	✗	✗	✗	✗	✓

**Table 2:** Toolchain used in the experiment.

Tool Components	Version/Configuration	Description
Solidity	0.8.28	Smart contract development language
Hardhat	^2^.x	Local development and deployment framework
Node.js	≥v16	JavaScript execution environment
Ethers.js	^5^.x	Interaction with blockchain, signature verification
Open Zeppelin	^4^.x	Security library (ECDSA)

## Data Availability

The data presented in this study are available in the article.

## Abbreviations

The following abbreviations are used in this manuscript:

CBDC	Central Bank Digital Currency
NFC	Near Field Communication
TEEs	Trusted Execution Environments
PW	Pure Wallet
HD	Hierarchical Deterministic
SE	Secure Element
ECDSA	Elliptic Curve Digital Signature Algorithm

## References

[1] Zheng Z., Xie S., Dai H.N., Chen X., Wang H. (2018). Blockchain challenges and opportunities: A survey. Int. J. Web Grid Serv..

[2] Keister T., Sanches D. (2023). Should central banks issue digital currency. Rev. Econ. Stud..

[3] Espedal S.B., Janzso D.A. (2022). Design Choices for Offline Transactions in a Norwegian Central Bank Digital Currency. Ph.D. Thesis.

[4] Ozili P.K. (2023). Central bank digital currency research around the World: A review of literature. J. Money Laund. Control.

[5] Chu Y., Lee J., Kim S., Kim H., Yoon Y., Chung H. (2022). Review of offline payment function of CBDC considering security requirements. Appl. Sci..

[6] Christodorescu M., Gu W.C., Kumaresan R., Minaei M., Ozdayi M., Price B., Raghuraman S., Saad M., Sheffield C., Xu M. (2020). Towards a two-tier hierarchical infrastructure: An offline payment system for central bank digital currencies. arXiv.

[7] Aboulaiz L., Akintade B., Daud H., Lansey M., Rodden M., Sawyer L., Yip M. (2024). Offline Payments: Implications for Reliability and Resiliency in Digital Payment Systems.

[8] Atangana O., Barbier M., Khoukhi L., Royer W. Securing Privacy in Offline Payment for Retail Central Bank Digital Currency: A Comprehensive Framework. Proceedings of the Blockchain and Cryptocurrency Conference.

[9] Kumar A., Sah B.K., Mehrotra T., Rajput G.K. A review on double spending problem in blockchain. Proceedings of the 2023 International Conference on Computational Intelligence and Sustainable Engineering Solutions (CISES).

[10] Benjelloun S., El Aissi M.E.M., Loukili Y., Ben Ali S.W., Chougrad H., El Boushaki A. Big Data Processing: Batch-based processing and stream-based processing. Proceedings of the 2020 Fourth International Conference on Intelligent Computing in Data Sciences (ICDS).

[11] Tripathi M., Mukhopadhyay A. (2020). Financial loss due to a data privacy breach: An empirical analysis. J. Organ. Comput. Electron. Commer..

[12] Hossain A., Rahaman H., Jamil A., Khan M.A. (2020). An algorithm for securing user credentials by combining Encryption and Hashing method. Int. J. Electr. Eng. Appl. Sci. (IJEEAS).

[13] Anwar M.R., Apriani D., Adianita I.R. (2021). Hash algorithm in verification of certificate data integrity and security. APTISI Trans. Technopreneurship (ATT).

[14] Bhatnagar S., Dayal M., Singh D., Upreti S., Upreti K., Kumar J. (2023). Block-hash signature (BHS) for transaction validation in smart contracts for security and privacy using blockchain. J. Mob. Multimed..

[15] Attarde K., Jaiswal C., Khatwani R., Pinto G., Kumar V. (2025). A novel central bank digital currency framework design for offline and foreign transactions based on blockchain. Digit. Policy Regul. Gov..

[16] Minwalla C., Miedema J., Hernandez S., Sutton-Lalani A. (2023). A Central Bank Digital Currency for Offline Payments.

[17] Thakur S., Breslin J.G. Real-time Peer to Peer Energy Trade with Blockchain Offline Channels. Proceedings of the 2020 IEEE International Conference on Power Systems Technology (POWERCON).

[18] San A.M., Sathitwiriyawong C. Privacy-preserving offline mobile payment protocol based on NFC. Proceedings of the 2016 International Computer Science and Engineering Conference (ICSEC).

[19] Michalopoulos P., Olowookere O., Pocher N., Sedlmeir J., Veneris A., Puri P. (2025). Privacy and Compliance Design Options in Offline Central Bank Digital Currencies. IEEE Trans. Netw. Serv. Manag..

[20] Abla P., Li T., He D., Huang H., Yu S., Zhang Y. (2024). Fair and Privacy-Preserved Data Trading Protocol by Exploiting Blockchain. IEEE Trans. Inf. Forensics Secur..

[21] Jie W., Qiu W., Koe A.S.V., Li J., Wang Y., Wu Y., Li J., Zheng Z. (2024). A Secure and Flexible Blockchain-Based Offline Payment Protocol. IEEE Trans. Comput..

[22] Wu F., Ye A., Diao Y., Zhang Y., Chen Y., Huang C. (2025). TrustChain: A privacy protection smart contract model with Trusted Execution Environment. Blockchain Res. Appl..

[23] Fang F., Ventre C., Basios M., Kong H., Kanthan L., Li L., Martinez-Rego D., Wu F. (2022). Cryptocurrency trading: A comprehensive survey. Financ. Innov..

[24] Igboanusi I.S., Dirgantoro K.P., Lee J.M., Kim D.S. (2021). Blockchain side implementation of pure wallet (pw): An offline transaction architecture. ICT Express.

[25] Wang H., Li X., Gao J., Li W. (2019). MOBT: A kleptographically-secure hierarchical-deterministic wallet for multiple offline Bitcoin transactions. Future Gener. Comput. Syst..

[26] Chow S.S.M., Choo K.K.R., Han J. (2021). Editorial for accountability and privacy issues in blockchain and cryptocurrency. Future Gener. Comput. Syst..

[27] Ivanov N., Yan Q. (2021). System-wide security for offline payment terminals. International Conference on Security and Privacy in Communication Systems.

[28] Dmitrienko A., Noack D., Yung M. Secure wallet-assisted offline bitcoin payments with double-spender revocation. Proceedings of the 2017 ACM Asia Conference on Computer and Communications Security.

[29] Takahashi T., Otsuka A. (2019). Short paper: Secure offline payments in bitcoin. International Conference on Financial Cryptography and Data Security.

[30] Li R., Wang Q., Zhang X., Wang Q., Galindo D., Xiang Y. An Offline Delegatable Cryptocurrency System. Proceedings of the 2021 IEEE International Conference on Blockchain and Cryptocurrency (ICBC).

[31] Kurt A., Sahin A., Harrilal-Parchment R., Akkaya K. LNMesh: Who Said You need Internet to send Bitcoin? Offline Lightning Network Payments using Community Wireless Mesh Networks. Proceedings of the 2023 IEEE 24th International Symposium on a World of Wireless, Mobile and Multimedia Networks (WoWMoM).

[32] Yang B., Feng W., Qin Y., Zhang Y., Tong D. (2024). Dual Offline Anonymous E-payment Scheme for Mobile Devices Based on TEE and SE. J. Softw..

[33] Yang B., Zhang Y., Tong D. DOPS: A Practical Dual Offline Payment Scheme of CBDC for Mobile Devices. Proceedings of the 2023 IEEE 22nd International Conference on Trust, Security and Privacy in Computing and Communications (TrustCom).

[34] Yang B., Zhang Y., Tong D. (2022). DOT-M: A dual offline transaction scheme of central bank digital currency for trusted mobile devices. International Conference on Network and System Security.

[35] Cecchetti E., Zhang F., Ji Y., Kosba A., Juels A., Shi E. Solidus: Confidential distributed ledger transactions via PVORM. Proceedings of the 2017 ACM SIGSAC Conference on Computer and Communications Security.

[36] Ahamad S.S. (2022). A Novel NFC-Based Secure Protocol for Merchant Transactions. IEEE Access.

[37] Ebadi Ansaroudi Z., Sharif A., Sciarretta G., Marino F.A., Ranise S. (2025). Secure and Reliable Digital Wallets: A Threat Model for Secure Storage in eIDAS 2.0. IFIP Annual Conference on Data and Applications Security and Privacy.

[38] Caudevilla O., Kim H.M. (2022). The Digital Yuan and Cross-Border Payments: China’s Rollout of Its Central Bank Digital Currency.

[39] Huibers F. (2024). Distributed ledger technology and the future of money and banking: Banking is necessary, banks are not. Bill Gates 1994. Account. Econ. Law Conviv..

[40] Si H., Huang Y., Li G., Zhao Y., Qi Y., Chen W., Gao Z. (2026). A Cryptocurrency Dual-Offline Payment Method for Payment Capacity Privacy Protection. Electronics.

